# Emerging Recombinant Oncolytic Poliovirus Therapies Against Malignant Glioma: A Review

**DOI:** 10.7759/cureus.34028

**Published:** 2023-01-21

**Authors:** Onkar R Dighe, Paresh Korde, Yuganshu T Bisen, Sandeep Iratwar, Anukriti Kesharwani, Sauvik Vardhan, Abhinesh Singh

**Affiliations:** 1 Department of Neurosurgery, Jawaharlal Nehru Medical College, Datta Meghe Institute of Higher Education and Research, Wardha, IND

**Keywords:** malignant, oncolytic, neuroectodermal, stem cells, necl-5, glioblastoma, poliovirus

## Abstract

Glioblastoma multiforme (GBM) is a fourth-grade malignant glioma that continues to be the main contributor to primary malignant brain tumour-related death in humans. The most prevalent primary brain tumours are gliomas. The most dangerous of these neoplasms, GBM, has been shown to be one of the most lethal and refractory tumours. For those who have been diagnosed with GBM, the median time to progression, as determined by magnetic resonance imaging, is roughly six months, and the median survival is approximately one year. GBM is challenging to manage with old treatments like chemotherapy, tumour debulking, and radiation therapy. Treatment outcomes are poor, and due to this effect, the treatment is not up to the mark. GBM also shows diagnostic complexity due to limitations in the use of specific targeted therapies. The treatment protocol followed currently has an entire focus on safe resection and radiotherapy. Protein synthesis is not tightly regulated physiologically in malignant cells, which promotes unchecked growth and proliferation. An innovative, experimental technique for treating cancer uses polioviruses that have been genetically altered to target a fascinating aberration of translation regulation in cancer. This approach enables precise and effective cancer cell targeting based on the convergence of numerous variables. Oncolytic viruses have revolutionised cancer treatment. However, their effectiveness in glioblastoma remains restricted, necessitating more improvement. Oncolytic poliovirus has shown great potential in the treatment of GBM. Factors like the blood-brain barrier, immunosuppressive tumour microenvironment (TME), and tumour heterogeneity make treatment for malignant gliomas ineffective. In this review, we have focused on oncolytic viruses, specifically oncolytic poliovirus, and we explore malignant glioma treatments. We have also discussed currently available conventional treatment options for malignant glioma and other brain tumours.

## Introduction and background

Astrocytoma, oligodendroglioma, and oligoastrocytoma are gliomas that originate from glial or progenitor cells [1,2]. Gliomas are categorised by the World Health Organisation (WHO) into four classes. Grade one and two gliomas are low grade, and grade three and four gliomas are high grade [3]. High-grade gliomas have a dismal prognosis. A median 11.6-year survival rate is observed in low-grade gliomas [4]. Glioblastoma multiforme (GBM) is the most prevalent grade four glioblastoma. The Food and Drug Administration has only licenced a few medications to treat glioma. The blood-brain barrier (BBB) in the central nervous system (CNS) that blocks many anticancer medications from accessing the brain is the cause of the delayed development in various treatment modalities [5,6]. The great majority of ectodermal/neuroectodermal tumors that contain the cellular receptor of poliovirus are naturally targeted by the virus. It may be linked to cancer cell populations that resemble stem cells and growing tumor vasculature. Poliovirus peculiar protein synthesis initiation mechanism is used to promote the translation of virus, spread, and cytotoxicity in glioblastoma. The poliovirus has been genetically modified to multiply and destroy malignant cells only when regulatory sequences from human rhinovirus type 2 (HRV-2) are introduced. For poliovirus derivatives to effectively treat cancer, targeted tumors must include a cluster of differentiation-155 (CD155). The most frequent malignant primary brain tumor is GBM, with 3.19 occurrences per 100,000 persons per year. With only a 4-5% five-year survival rate and a meager 26-33% two-year survival rate in therapy studies, GBM has a rather bad prognosis. Initially, there were reports of remissions of cancer that coincided with infection caused naturally or viruses that precisely target malignant cells for elimination served as the inspiration for the concept of oncolytic viruses [7].

Viral-based antineoplastic methods can now be customized thanks to developments in our molecular processes behind cell type selectivity, cytotoxicity, and viral host cell tropism. Current attempts to employ viruses treatment of cancer are based on orphan reovirus animal diseases [8,9] or human pathogens that are modified genetically to preferentially destroy malignant cells while producing little cytotoxicity in surrounding normal cells like Herpes simplex virus-1 (HSV-1) [10]. Replication-competent viruses are used in oncolytic virotherapy to target and destroy malignant cells. Apoptosis, pyroptosis, and necroptosis are a few of the numerous methods by which oncolytic viruses cause the death of cancer cells. Tumour-associated antigens are viral pathogens that are related to molecular patterns. Direct oncolysis triggers an immune response in the tumour microenvironment (TME), which is an inflammatory type. Malignant glioma and other tumours that spread to the CNS have highly immunosuppressive TME tolerant T-cells against tumour-specific antigens, T-cell deletion, and systemic immunosuppression brought on by T-cell sequestration in the bone marrow are all contributing factors to the local and systemic immune suppression within and around malignant tumour glioma.

## Review

Methodology

**Figure 1:**
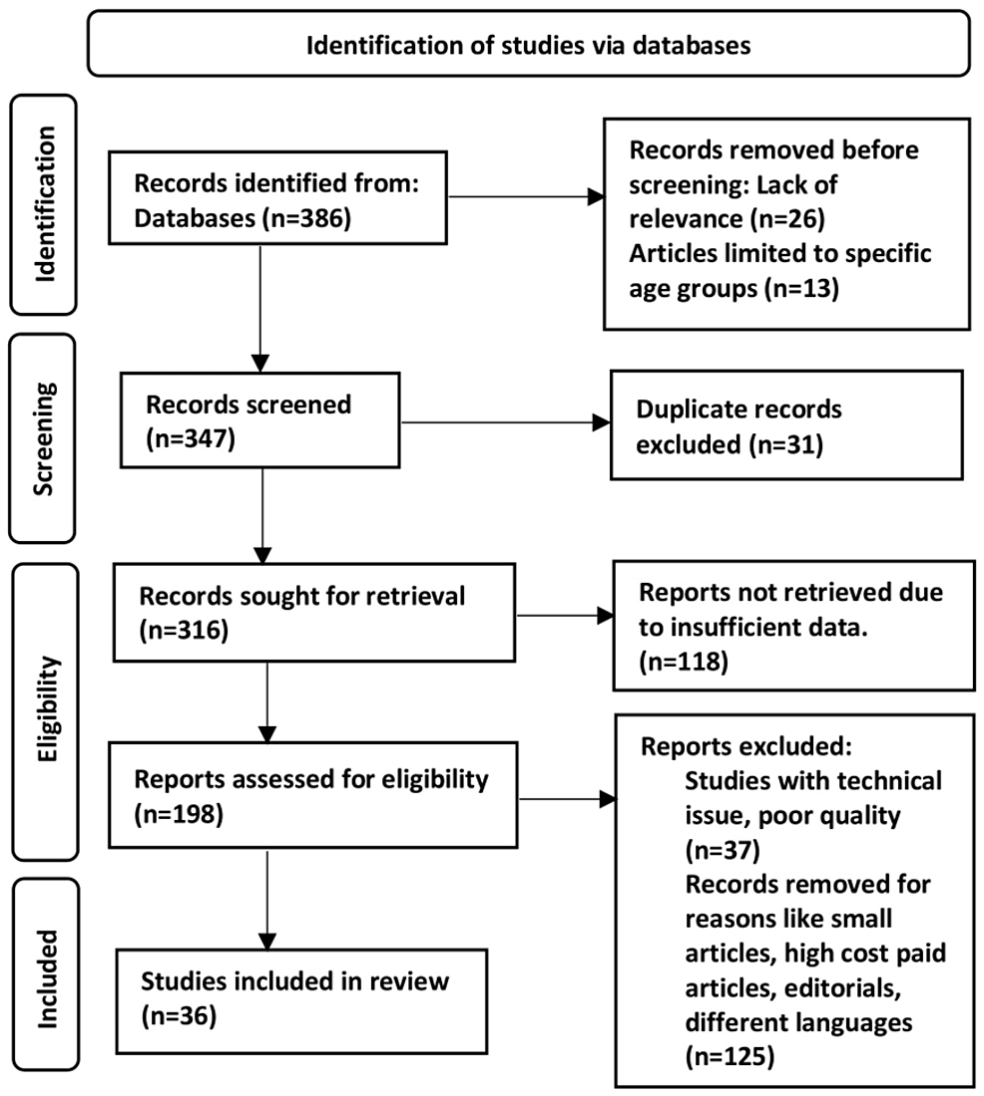
PRISMA model for the search strategy PRISMA: Preferred Reporting Items for Systemic Reviews and Meta-Analyses

Google Scholar, Embase, and advanced PubMed search were used to search the following keywords: glioblastoma multiforme AND oncolytic virus OR poliovirus AND neuroectodermal. The search yielded 386 articles, of which 36 research publications were selected for research. The methodology by Preferred Reporting Items for Systemic Reviews and Meta-Analyses (PRISMA) method is shown in Figure 1.

Treatment options available currently

There are various treatment options like surgery and chemoradiation, chimeric antigen receptor T-cell therapy, Immunotherapy, vaccine therapy, and oncolytic virotherapy that includes oncolytic poliovirus, oncolytic adenovirus, etc. Present treatment options are mainly conventional and they have serious side effects on healthy tissues of the body such as radiation exposure. In the current scenario, the paediatric age groups having malignant glioma are treated with intensive multidrug therapy cycles and radiation therapy. Despite the aggressive approach in treatment modalities, recurrence and many ill effects are there. We will discuss a recombinant oncolytic poliovirus-based cancer therapy in this article. The main goal of this review is to define the biological determinants of viral tumour tropism and target cell death to facilitate the logical design of clinical research. We will concentrate on oncolytic poliovirus recombinants in this article because they have minimal mechanistic similarities to other suggested oncolytic virus species.

Oncolytic poliovirus: mechanism against tumour cells and its efficacy

A unique feature of the oncolytic poliovirus is its replication-competent behaviour [10]. It propagates efficiently in tumours but is highly restricted to target tissues, which is the most useful feature for oncolytic tumour therapy. The strategy outlined here is intended to have the dual benefits of directly destroying tumour cells and activating the host immune system. Poliovirus infection causes rapid cell death and lysis in susceptible transformed cells. While several early viral life cycle events may be involved, the production of the viral 2A protease (2Apro) is a crucial process which leads to host cell death. To carry out the effect of poliovirus to suppress the gene expression mechanism of host cells and promote translation of viral content, 2Apro rapidly cleaves vital elements of host cells involved in the export of mRNA [11] and the process of translation [12]. 2Apro is sufficient to cause cell death, demonstrating its powerful cytolytic capabilities [13]. The poliovirus has a concise life cycle because of its RNA structural component. Instead of developing complex parasitic interactions with its host cells, the poliovirus quickly overtakes and destroys them to maximise its ability to spread. Translating viral proteins can begin as soon as the virus DNA is exposed. Because it is the primary method used by the poliovirus to anticipate the host cell's defensive responses, this step is most important in its life cycle. We can call it the rate-limiting step.

The viral 2Apro is the first nonstructural polypeptide produced by the poliovirus. Effects of 2Apro proteolysis cause host cell gene expression to be shut down, which limits antiviral responses that call for protein synthesis. Strong host defences against infected or lysed tumours are produced due to the short and damaging interactions of the virus with host cells. A significant amount of work was put into defining tumour cell targeting mechanisms at the molecular level, elucidating the molecular mechanisms causing selective tumour cytotoxicity, and producing poliovirus recombinants that are non-pathogenic [14,15]. Cancer cell death via intra-tumoral viral injection with tumour-selective cytotoxicity may reduce neoplastic lesions. It is possible to directly kill cancer cells by invading neutrophils and other immune populations in response to localised, virus-induced inflammation. This operation may be essential for enhancing anti-tumour immunity and significantly slowing the spread of cancer cells [16,17]. The paradoxical promotion and restriction of tumour development by tumour-associated neutrophils are similar to that of macrophages. On the other hand, it is thought that after contracting a pathogen, neutrophils engage in inflammatory and cytotoxic processes that are detrimental to the growth of tumours. Figure 1 summarises the mechanism of oncolytic poliovirus.

**Figure 2:**
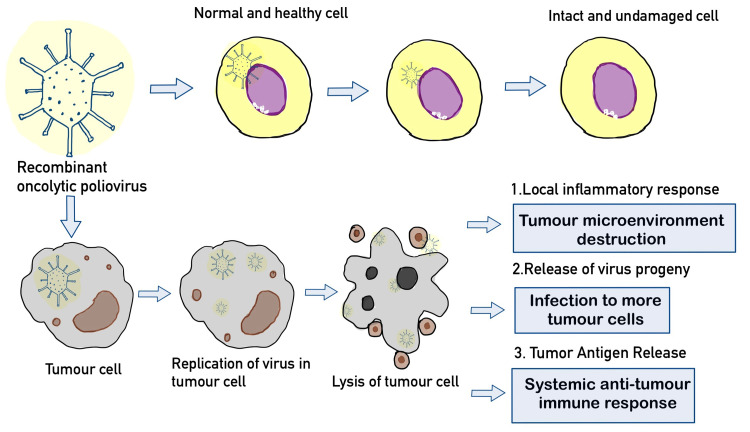
Mechanism of recombinant oncolytic poliovirus against GBM tumour cells GBM: Glioblastoma multiforme Source: Original

Internal ribosomal entry sites (IRES)

In the Picornaviridae family, single-stranded RNA-encapsulated viruses include the neurotoxic poliovirus, which is notable for its effects [18]. The internal ribosome entry site (IRES) of the Sabin (live attenuated poliovirus type 1) has been swapped out for human rhinovirus type two in the prototype oncolytic poliovirus created [19]. It is found that the IRES is a crucial pathogenic determinant before considering the approach outlined here. By replacing the poliovirus IRES with its counterpart from HRV2, polio/rhinovirus chimaera, the poliovirus's capacity to translate, grow, and kill spinal cord motor neurons was completely eliminated. The IRES of the poliovirus genome, five untranslated regions, and, more specifically, two nearby dual stem-loop structures inside the IRES have been linked to neuropathogenicity [20].

Recombinant nonpathogenic polio-rhinovirus chimera (PVSRIPO)

Oncolytic poliovirus, also known as PVSRIPO, was produced by swapping the original internal ribosome entry site from HRV2. PVSRIPO has been shown to be highly safe and effective in various tumour types, including malignant glioma. This IRES gene has mutations that correspond to domain V in the Sabin poliovirus strains used for immunisation. Despite the fact that the poliovirus and HRV2 IRES elements are physically closely related, it has been demonstrated that replacing the whole element with a non-pathogenic form from HRV2 attenuates the virus without compromising its capacity to thrive in non-neuronal cells. The intergeneric poliovirus CD155 receptor with the HRV2 IRES is known as prototype intergeneric poliovirus chimera PV1 (RIPO) which exhibits reduced replication in neural cells and does not result in poliomyelitis [21-23]. Intratumoral PV1 (RIPO) injection is necessary for malignant gliomas to demonstrate anti-tumour activity. This was demonstrated in Nectin-like molecules-5 (Necl-5) in a number of neuron-like cell lines, including human embryonic kidney 293 (HEK293) cells and neuroblastoma cells. The attenuation of RIPO was maximised by PVSRIPO. Serotype one live attenuated Sabin poliovirus vaccine containing the HRV2 IRES is sold under the trade name PVSRIPO. Table 1 summarises the development of recombinant polioviruses.

**Table 1:** Development of various recombinant oncolytic poliovirus PV: poliovirus; PV1 (RIPO): prototype intergeneric poliovirus chimera; PVSRIPO: recombinant nonpathogenic polio-rhinovirus chimera; IRES: internal ribosomal entry site; HRV2: human rhinovirus serotype 2 Source: The table was created from information in Gromeier et al. (1996) [19]

Sr. No.	Recombinant Oncolytic Poliovirus	Description
1	PV1 (RIPO)	It is the original chimera prototype, designed with the complete genome of wild type of PV type 1 with IRES replaced with HRV2
2	PVSRIPO	It contains the whole genome of PV1 Sabin vaccine strain with IRES region replaced with HRV2
3	PV1 (RIPOS)	It substituted the capsid coding area of PV type 1 Sabin vaccination strain with the capsid coding region (P1)

Studies and Clinical Trials Related to PVSRIPO

According to a study, cynomolgus macaques were administered intracerebrally in additional studies examining the toxicity, biodistribution, and shedding of PVSRIPO, but no sickness nor mortality was noted. Despite having been discovered to be neuronally incompetent, in vitro research showed that this polio-rhinovirus chimaera was capable of infecting glioma cells, decreasing their strength, and causing cytolysis in glioma [24,25]. PVSRIPO could prolong longevity and stop tumour growth in GBM. Its effectiveness was discovered to be associated with the over-expression of CD155. In phase one of a clinical trial, intra-tumoral infusion of PVSRPO in patients with a recurrent type of grade four malignant glioma revealed low neurovirulence, and patient rate of survival was greater. Sixty-one patients having WHO grade four glioblastoma, which was a recurrent type, were infused using PVSRIPO as part of the first phase of the clinical trials. As a result of its modest efficacy in the trial, the findings not only confirmed the success of intratumoral viral delivery in people but also showed improvement in the survival rate of the patients. Initially, the survival rate at 24 months and 36 months was 21% with variability in the survival period of 70, 69 and 57 months being observed after PVSRIPO infusion [26-28].

Relation of CD155 with oncolytic viruses

Numerous interactions between oncolytic viruses and the host cell affect their ability to combat cancer. Cellular receptors are of utmost importance for viral entry in certain types of malignant cells for oncolysis. The immunoglobulin superfamily (IGSF) member CD155 appears to be the only cell-surface molecule that allows the poliovirus to enter cells [29]. Studies of neuroectodermal tumour cell lines that respond to oncolytic poliovirus-based treatment provide the majority of the data for CD155 expression in these tumours. High-grade malignant glioma is usually correlated with CD155 overexpression. The levels of CD155 expression in tumour tissues matched those in primary tissue cultures produced from the tumours. Due to its relationship with malignancies associated with neuroectodermal origin, CD155 ligand poliovirus is best for developing oncolytic viral therapies targeting glioma cells. Poliovirus susceptibility is mediated by CD155, which is both necessary and sufficient. Expression of CD155 develops typical paralytic poliomyelitis after viral infection, in contrast to their wild-type counterparts [30]. Malignant glioma cells are vulnerable to PVSRIPO because of the critical function that CD155 plays in poliovirus susceptibility. The CD155-ligand poliovirus is an excellent choice for the development of oncolytic viral therapeutics against cancerous glioma cells due to its association with neuroectodermal malignancies. CD155 plays a magnificent role in the entry and the overall action of recombinant poliovirus against tumour cells. The fact that cancer-related transcription factors control the expression of the CD155 gene may indicate that these elements are involved in a tumour's expression [31].

Necl-5 tumour antigen and its relation with tumour cells

The poliovirus receptor, known as Necl-5, was first discovered using antibodies produced against fractionated HeLa cell membranes that were virus-neutralising [32,33]. Necl-5 is potent enough, according to all current empirical evidence, to make mammalian cells susceptible to wild type of poliovirus. Due to the virus's dependence on Necl-5, the poliovirus only infect humans and old-world primates. Exogenous human Necl-5 can be used to get around this restriction. For instance, after contracting the poliovirus, transgenic mice start expressing the human Necl-5 gene and manifest a condition resembling the paralytic type of poliomyelitis in human beings. Patterns of expression of Necl-5 and poliovirus susceptibility overlap in vulnerable primates. For instance, cells present in the epithelial lining and structures associated with lymphatics, which are currently unknown cell types, express Necl-5 at the initial viral infection site in the gastrointestinal tract [34]. The nectin-like genes include extracellular cell adhesion molecules with three immunoglobulins. It also contains a cytoplasmic domain and a transmembrane region. The variety of adhesion of cells has been linked to Necl molecules in several crucial pathways, which are physiological, such as tissue responses during development or regeneration. The members of the Necl domain serve as the components that let herpes simplex viruses connect to cells, in addition to its function as a receptor for the poliovirus [35].

Although the objective biological function is unknown, Necl-5 can be expressed in CNS during the embryonic period and play an essential role in developing the CNS structures like other Necl molecules. Necl-5 is widely linked to malignancy, in contrast to the mature organism's restricted expression. There may be the influence of molecule's expression on the essential characteristics of malignant cells like metastasis, invasion and contact inhibition and unbalanced proliferative regulations. Expression of Necl-5 is observed in all the cell lines, which are derived from neuroectodermal malignant conditions, except for some lymphoid cell lines [36]. Some reports suggest that glial, colorectal, hepatocellular, breast, and lung carcinomas exhibit the same.

Immunohistochemistry examinations done on tumours from GBM patients confirmed the extensive Necl-5 expression, sensed by Immunoblot of lysates obtained from similar tumours. These experimental studies also discovered that CD133+ stem cell-like GBM cells were strongly expressed in patient populations and the developing tumour vasculature. For effective targeting of GBM, the presence of Necl-5 in such major tumour components may be especially crucial. Apart from specific molecular factors like the expression of Necl-5 in glioblastoma, these tumours make a clear and attractive target for oncolytic poliovirus therapy. The prognosis for people with GBM is particularly dismal because of the absence of efficient treatments. Many more traditional modes of treatment may be especially amenable to generating anti-tumour responses when exposed to poliovirus.

## Conclusions

In this article, we have discussed different treatment options for tumours and the benefits of recombinant poliovirus in glioma. Conventional treatment options are not up to the mark and oncolytic viruses show great potential in the treatment domain. Certain types of glioma, like GBM, are hard to treat, and they offer good outcomes when oncolytic virus treatment modalities are used with standard treatment protocols. Viral replication, penetration and infiltration of tumours, cytotoxic effects, and apoptotic effects of oncolytic poliovirus have proven to be adequate. We have discussed the efficacy of oncolytic poliovirus and its mechanism along with PVSRIPO, Necl-5, CD155, and their relation with glioblastoma. Oncolytic viral medicines that target human cells provide major advantages over radiation therapy, which has severe side effects on non-cancerous tissues. A significant development in genetic engineering to malignancies, human clinical trials are adding value to this treatment modality. Total dependence on oncolytic virus therapy as the standard treatment method for gliomas requires extensive research. This review strongly supports the use of recombinant oncolytic poliovirus as a treatment modality in cases of malignant glioma. Clinical trials using oncolytic poliovirus have given some good outcomes. In the upcoming era, comparative studies of treatment results and survival rates with other viruses and other modalities are also required to determine the most significant potential.
